# Resonance Raman Spectroscopy and Density Functional Theory Reveal the Hemin Release Mechanism of Fish and Mammalian Hemoglobin

**DOI:** 10.1021/acs.jafc.5c12506

**Published:** 2026-02-20

**Authors:** Sean M. Baker, Ryan L. Hall, Thomas C. Brunold, Mark P. Richards

**Affiliations:** † Department of Food Science, University of Wisconsin−Madison, Madison, Wisconsin 53706, United States; ‡ Department of Chemistry, University of Wisconsin−Madison, Madison, Wisconsin 53706, United States; § Department of Animal and Dairy Sciences, University of Wisconsin−Madison, Madison, Wisconsin 53706, United States

**Keywords:** hemin dissociation, resonance Raman, DFT computations

## Abstract

Salmonids are an incredibly valuable agricultural commodity, with large market growth expected over the next 10 years. Salmonids have a high feed conversion ratio and are rich in vital nutrients; however, their post-mortem tissues are subject to deterioration due in part to their labile hemoglobin (Hb). In this work, we aimed to understand the driving force for the pro-oxidative nature of salmonid (trout IV) Hb. We utilized resonance Raman spectroscopy (rR), electronic absorption spectroscopy (EA), and density functional theory (DFT) calculations to probe the distal and proximal heme pocket architectures of trout IV and bovine Hb. Using fluoride as a H-bond-sensitive ligand, we found that at low pH, trout IV ferric Hb is more likely to have a protonated distal His. The enhanced distal His protonation and mobility play a crucial role in hemin dissociation, explaining the oxidative capacity of salmonid Hb, which dwarfs that of mammalian Hb.

## Graphical abstract

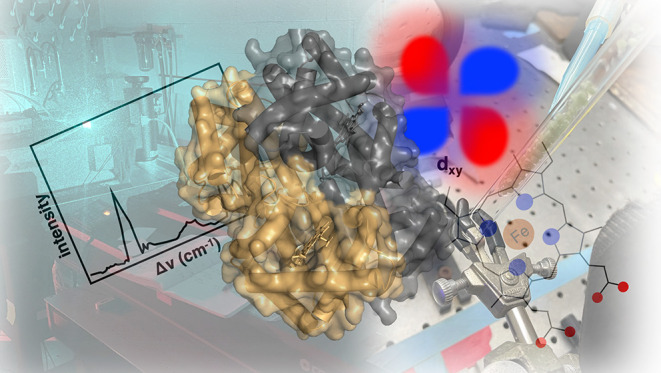

## Introduction

Muscle foods from salmonids (trout and salmon) are high-quality sources of protein and long-chain, highly unsaturated fatty acids (e.g. EPA, DHA) for consumers.^1,2^ The 2024 US trout and salmon market sizes were estimated at ∼$324M^3^ and ∼$1,479M,^4^ respectively, with substantial increases in market value forecasted in the coming years. Even more, salmonid farming has been made substantially more accessible (reduced food miles, in-land production) and more sustainable (recirculating water systems) through the advent of aquaculture.^5^ Given their nutritional and sustainability attributes, salmonids will be a vital agricultural commodity in the future. However, the high concentration of long-chain, highly unsaturated fatty acids in salmonids leads to rapid lipid and protein oxidation, resulting in organoleptic changes such as off-odors, off-flavors, and color deterioration.^6^

One of the major drivers of these degradation processes is hemoglobin (Hb),^7^ the iron-containing oxygen-transport protein housed within red blood cells (RBCs). Despite fish processors’ best attempts to bleed the fish, it is impossible to remove all residual RBCs within the capillaries surrounding myocytes and adipocytes. Post-mortem changes (i.e., pH drop, osmotic pressure, freeze–thaw) in the tissue will cause the RBCs to burst, releasing pro-oxidant Hb.^8,9^ The Hb contents of post-mortem bovine and trout muscle are ∼0.5 mg/g^10–16^ and 1.1 mg/g,^17^ respectively. The Hb iron can then undergo pH-mediated redox changes and hemin (ferric) dissociation, with the latter being critical for lipid oxidation pathways.^18^ The propensity to release hemin in the post-mortem muscle environment is far more pronounced in salmonid Hb compared to bovine Hb.^19,20^ However, the precise mechanism governing the elevated hemin release of salmonid Hb is unclear. It is almost certain that hemin release is, in part, governed by the activity of the distal (HisE7) and heme-coordinating proximal (HisF8) histidine residues. Previous work with carboxy sperm whale myoglobin (Mb) has suggested that a reduction in pH (<6) leads to a large fraction of HisE7 to adopt an “open” configuration.^21^ The open configuration may decrease distal water occupancy, a pH-dependent phenomenon that has been observed in ferrous myoglobin.^22^ This event is driven by the loss of the H-bond between the HisE7 residue and distal water, as evidenced by decreased distal water occupancy observed by X-ray crystallography following the replacement of HisE7 with a non-H-bonding amino acid.^23^ In the ferric sperm whale Mb variants H64V and H64L, which lack water at the sixth coordination site,^24^ the rate of hemin release increases by 7.3- to 11*-fold* relative to wild-type Mb.^25^ This begs the question of whether salmonid Hb possesses a more open distal heme pocket that results in a weaker coordination of the distal water ligand.

During hemin release, there is also a change in the nature of the proximal coordination, particularly the Fe–N_prox_ bond length. Hargrove et al.^26^ found that an increase in the Fe–N_prox_ bond length led to an increase in hemin release. However, in addition to differences in HisE7 protonation, it is possible that salmonid Hb presents a longer HisF8-iron bond that contributes to hemin release.

To our knowledge, no researchers have compared the p*K*_a_ and proximal HisF8-iron bond strengths of salmonid and bovine Hb. In the present study, we utilized trout IV Hb because of its well-defined Root effect (low O_2_ affinity of Hb at low pH even at high O_2_ partial pressure),^27,28^ auto-oxidation (*k*_ox_),^29^ hemin dissociation,^29^ and lipid oxidation capacity.^19^ The cathodic fraction (Hb I–III) of rainbow trout was not examined primarily due to its 15× increased hemin affinity compared to trout IV metHb.^30^ We employed resonance Raman (rR) spectroscopy, electronic absorption (EA) spectroscopy, and density functional theory (DFT) calculations to probe distal and proximal heme pocket differences between salmonid and bovine Hb.

## Materials and Methods

### Hemoglobin Isolation and Oxidation

Bovine and rainbow trout (*Oncorhynchus mykiss*) Hb were isolated from blood collected on the day of animal harvest, as outlined by Aranda et al.^29^ Briefly, fresh trout blood in 150 units/mL sodium heparin and 300 mM NaCl in 1 mM Tris, pH 8, was transported to the UW–Madison Meat Science and Animal Biologics Discovery building within 24 h of animal harvest. Bovine blood was collected following exsanguination. All animals were harvested according to the *Livestock in Meat Science Program* approved by the University of Wisconsin—Madison Institutional Animal Care and Use Committee (#A006389-R02). Plasma was removed via centrifugation (700×*g*, 4 min, 4 °C) and then discarded. The remaining RBCs were washed with 10 vol of 0.3 M NaCl in 1 mM Tris, pH 8, and then centrifuged, with the washing buffer discarded after each wash (washing was repeated 3×). After washing, RBCs were lysed for 1 h (2 °C) in 3 vol of 1 mM Tris, pH 8. Hb was then isolated from ghosts by the addition of 1/10 of the volume of 1 M NaCl, followed by centrifugation (28,000×*g*, 15 min, 4 °C). The Hb-rich supernatant was then desalted (Econo-Pac 10DG, Bio-Rad, Hercules, CA) into 1 mM Tris, pH 8, adjusted to 1 mM (heme basis, ε_415nm_ = 132 mM^–1^ cm^–1^), and stored at −80 °C. This concluded the purification process for bovine Hb. Rainbow trout hemolysate required further purification to isolate Hb isoform IV (trout IV Hb, ∼65% of trout hemolysate).^28^ Trout IV Hb was isolated from hemolysate using DEAE-52 resin equilibrated with 10 mM Tris at pH 8. Trout I–III Hb were eluted with 10 mM Tris, pH 8. Trout IV Hb was then eluted using 10 mM Tris, 0.5 M NaCl, pH 8, followed by buffer exchange into 10 mM Tris, 0.025 M NaCl, pH 8, and filtered through Superdex 200 using 10 mM Tris, 25 mM NaCl, pH 8.

Isolated Hb was predominant in the O_2_-bound oxy (Fe^2+^) state. Oxidation to metHb (Fe^3+^) was performed by the addition of potassium ferricyanide at 3× the molar heme concentration, followed by a 30 min reaction at 2 °C. Excess ferricyanide was removed via desalting into 1 mM Tris, pH 8. Hb was then adjusted to 1 mM (heme basis, ε_405nm_ = 153 mM^–1^ cm^–1^) and stored at −80 °C.

### MetHb-Fluoride Saturation

MetHb-Fluoride (metHb-F) saturation curves were calculated, as described by Geeraerts et al.,^31^ with minor modifications. The metHb stock was diluted to 38.6 μM in 100 mM sodium phosphate buffer, pH 6.0, and exposed to 0.05–500 mM NaF, and then analyzed by EA spectroscopy (350–700 nm) immediately following pipet aspiration of the reaction. Spectra were collected using a dual beam spectrophotometer (UV-2600, Shimadzu Corp., Kyoto, Japan) at room temperature in black matched cuvettes (ref cuvette contained buffer only), with a resolution of 1 nm and a scan rate of 17.5 nm/s. Saturation curves were made by plotting the fraction of metHb-F (r)[Fn fn1] vs [NaF] and fit with a Hill equation[Fn fn2] for cooperative binding. NaF saturation reactions monitored by EA were performed in duplicate.

### Resonance Raman Spectroscopy

For rR experiments, the heme concentration was 200 μM; thus, the [NaF] was increased to 360 mM based on our saturation experiments. Previous research using catalase has shown that high concentrations (500 mM) of NaF do not induce backbone perturbations as measured by circular dichroism.^32^ Furthermore, fluoride ligation to Hb and Mb limits hemin release,^33^ decreasing sample degradation during extended data collection. Taken together, we do not expect NaF to induce unwanted structural or functional changes on the Hbs being tested. Data were collected at pH 5.3 and pH 7.0, in each case with unique buffer compositions to minimize temperature-induced pH shifts.^34,35^ We selected a pH range in an attempt to capture HisE7 in different protonation states, with the low-end value (5.3) being slightly lower than the post-mortem pH of beef (∼5.5) and trout (∼6.1) skeletal muscle.^36,37^ The metHb stock from bovine or trout IV was diluted into buffer to a final concentration of 45 mM HEPES, 30 mM sodium phosphate, 0.25 mM Tris, 360 mM NaF (pH 7)^34^ or 80 mM MES, 50 mM sodium phosphate, 0.25 mM Tris, 360 mM NaF (pH 5.3).^35^ Following dilution, room-temperature pH values were verified (±0.03). MetHb-F solutions were then frozen, dropwise, in liquid N_2_ using a syringe equipped with a 22 Ga needle (Figure S1). To probe the proximal His–iron bond, ∼2 mM metHb samples in buffer pH 5.3 (same composition as above) were combined 50:50 (v/v) with glycerol to prevent ice crystallization.^38^ Samples in 50% glycerol were frozen as described above, with a final heme concentration of ∼1 mM (Figure S1).

rR spectra were obtained, as described previously,^39^ with a laser excitation wavelength (λ_ex_) of 514.5 or 457.9 nm, produced by a Coherent I-305 Ar^+^ laser. Laser power at the samples was maintained at ∼20 mW to avoid sample decomposition during the extended data collection. All data were collected at 77 K, using a quartz finger dewar loaded with sample beads in liquid N_2_. The 135° backscattered light was dispersed by a triple monochromator (SpectraPro -300i, Acton Research Corp., Acton, MA, USA) equipped with 1200 and 2400 grooves/mm gratings and analyzed with a deep-depletion, back-thinned, CCD camera (Spec. X-100 BR, Teledyne Princeton Instruments, Acton, MA, USA). The ice peak at 228 cm^–1^ was used as an internal standard. rR data were baseline-corrected using a two-point linear fit with the *line* interpolation method in Origin 2025. All spectra are the average of 3 readings from 1 sample, with a linear baseline correction applied for the viewing window (e.g., 325–700 cm^–1^).

### Computational Models

Density functional theory (DFT) calculations, as implemented in Orca 6.0,^40–45^ were employed to complement the spectroscopic data obtained in this study. Initial atomic coordinates of the heme moiety, distal His, proximal His, and the axial ligand were derived from a high-resolution (1.85 Å) crystal structure of bovine metHb (PDB ID: 2QSP).^29^ The heme ring was truncated by replacing the propionates with methyl groups, and a fluorine atom was placed in the axial position. To probe the effect of distal His protonation on the Fe–F bond properties, three different models were considered: (1) neutral distal imidazole with a proton on N_ε_ (HSE); (2) neutral distal imidazole with a proton on N_δ_ (ΗSD); and (3) positively charged distal imidazole with protons on both N_ε_ and N_δ_ (HSP). Geometry optimizations were performed spin-unrestricted using the PBE functional^46–48^ with the def2-TZVP basis set^49^ for the Fe, N, and F atoms, the def2-SVP basis set^50^ for all other atoms, and the auxiliary def2/J^51^ basis set. For the HSE and HSD models, the charge and spin multiplicity (2*S* + 1) were 0 and 6 (*S* = 5/2),^52^ respectively. For the HSP model, the charge and spin multiplicity were 1 and 6, respectively. The net charges were calculated as the sum of those of truncated protoporphyrin (−2), iron (+3), fluoride (−1), and HSE/HSD imidazole (0) or HSP imidazole (+1). To restrict the repositioning of the distal imidazole relative to the heme plane, the distal imidazole C_γ_ and all four heme C_m_ were fixed during the optimization of models containing a distal moiety.

In addition to fluorinated models, high-spin (HS, *S* = 5/2) and low-spin (LS, *S* = 1/2) truncated ferric heme models were evaluated. The HS model contained a truncated heme, distal (fixed) and proximal imidazoles (HSD), and an axial water ligand. The LS model contained a truncated heme with a distal and proximal imidazole (HSD), where the distal imidazole was oriented as the axial ligand (bis-His species). Geometry optimizations of these models were performed using the same functionals and basis sets as described above for the fluorine-bound models. Geometry constraints were applied to the distal imidazole C_γ_ and heme C_m_ to restrict the repositioning of the distal imidazole (only for the HS model, no constraints were set for the LS model). For the HS model, an additional optimization was performed to scan along the proximal Fe–His bond (Fe–N_prox_) using the same functional and basis sets as those in the initial optimization. Full optimization details for all models can be found in the Supporting Information.

### Frequency and TDDFT Calculations

Following geometry optimization, frequency calculations were performed with the same functional and basis sets as those described above. From the frequency calculations, Raman spectra were simulated using a 10 cm^–1^ peak broadening. Electronic transition energies and absorption intensities were calculated using the time-dependent DFT (TDDFT) method within the Tamm–Dancoff approximation^53^ using the same functional and basis sets as described above for the geometry optimizations. For each calculation, the first 160 transitions within an energy window of ± 3 *E*_*h*_ with respect to the HOMO/LUMO energies were considered. Full frequency and TDDFT inputs can be found in the Supporting Information. Vibrational eigenvectors were visualized in PyMol (v. 3.1.1) using the PyVibMS plugin.^54^

## Results

We set out to determine the protonation state of the distal His from trout IV and bovine ferric Hb at post-mortem pH (5.3) and near physiological pH (7.0). We used fluoride as a H-bond-sensitive probe^55^ and analyzed samples using rR spectroscopy to identify the iron-fluoride stretching frequency (νFe–F) at both pH levels. To ensure all heme sites of bovine and trout IV metHb were bound with F^–^, we performed a NaF titration to calculate the saturating concentration at intermediate pH (6.0) (Figure 1). Heme site saturation was tracked by the change in absorbance at 607 nm, which provided a saturating concentration of 134.4 and 135 mM for bovine and trout IV Hb, respectively. In addition to the increase in absorbance at 607 nm, increases at ∼571/∼476 nm and a decrease at ∼526 nm were observed with increasing F^–^ concentration. rR experiments required an increased heme concentration (200 μM); hence, the NaF concentration was increased to 360 mM.

**Figure 1:**
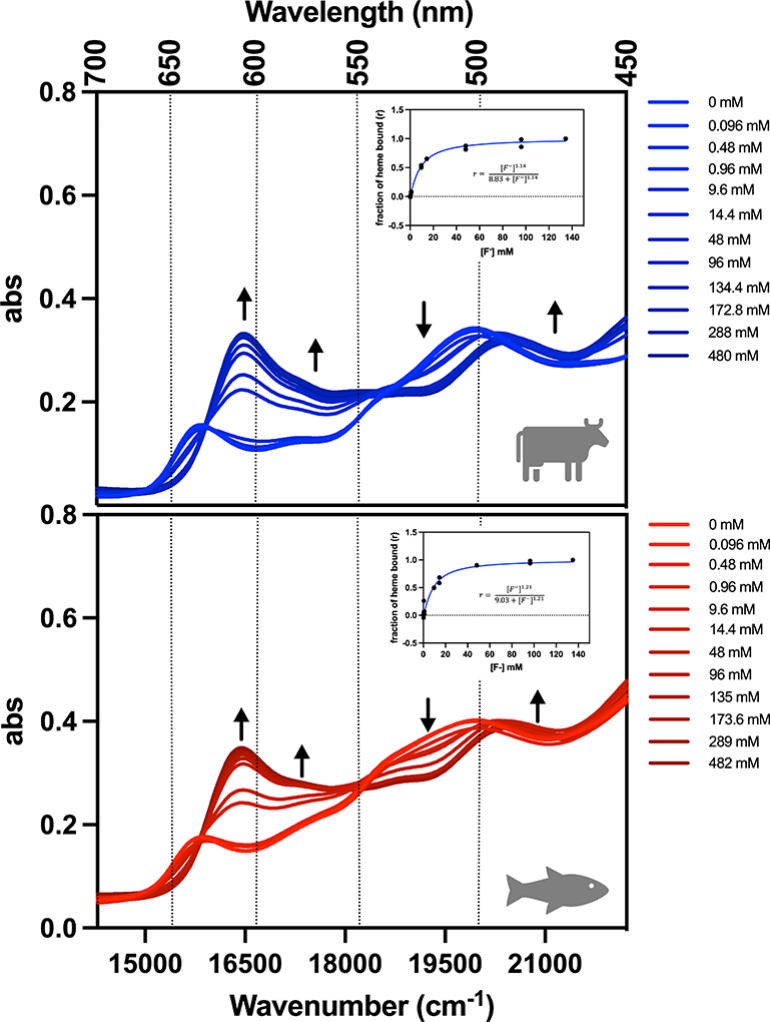
Bovine (top) and trout IV (bottom) methemoglobin (metHb, 38.6 μM heme basis) fluoride complex (metHb-F). Electronic absorption of metHb in the presence of increasing concentration of NaF. Arrow indicates the direction of change in absorption as the NaF concentration increases. Spectra were subject to smoothing for visualization. Concentrations listed on the inset are [NaF]. (Inset) Hill plot of NaF binding with the corresponding equation for cooperative binding. The saturation [F^–^] was 134.4 mM for bovine metHb and 135 mM for trout IV metHb. Buffer was 100 mM sodium phosphate, pH 6.0. The change in abs at 16474 cm^–1^ (607 nm) was used to calculate the fraction of heme bound (r). See the Materials and Methods section for full calculation.

Ahead of the rR analysis, room-temperature electronic absorption (EA) spectra of ferric hemoglobin fluoride (metHb-F) were collected (Figure 2A). Interestingly, following a reduction in pH from 7.0 to 5.3, a red shift of the lowest-energy charge transfer (CT1) transition located at ∼605 nm was much more pronounced for trout IV (+7 nm) compared to the bovine (+3 nm) metHb-F at low pH. Droghetti et al.^55^ stated that a CT1 red shift of ferric Hb-F complexes can be attributed to increased H-bond donation from distal residues to the fluoride ligand. With this notion in mind, the CT1 shift of trout IV metHb-F would be expected to occur at pH values higher than those for bovine metHb-F. In fact, this is what we observed when collecting EA spectra of metHb-F at pH levels between 7.0 and 4.8 (Figure 2B). A substantial CT1 shift was observed for trout IV metHb-F at pH 6.4, while an equivalent shift was not seen for bovine metHb-F until pH 5.8. This is the first piece of evidence suggesting a higher p*K*_a_ of the trout IV Hb distal His.

**Figure 2:**
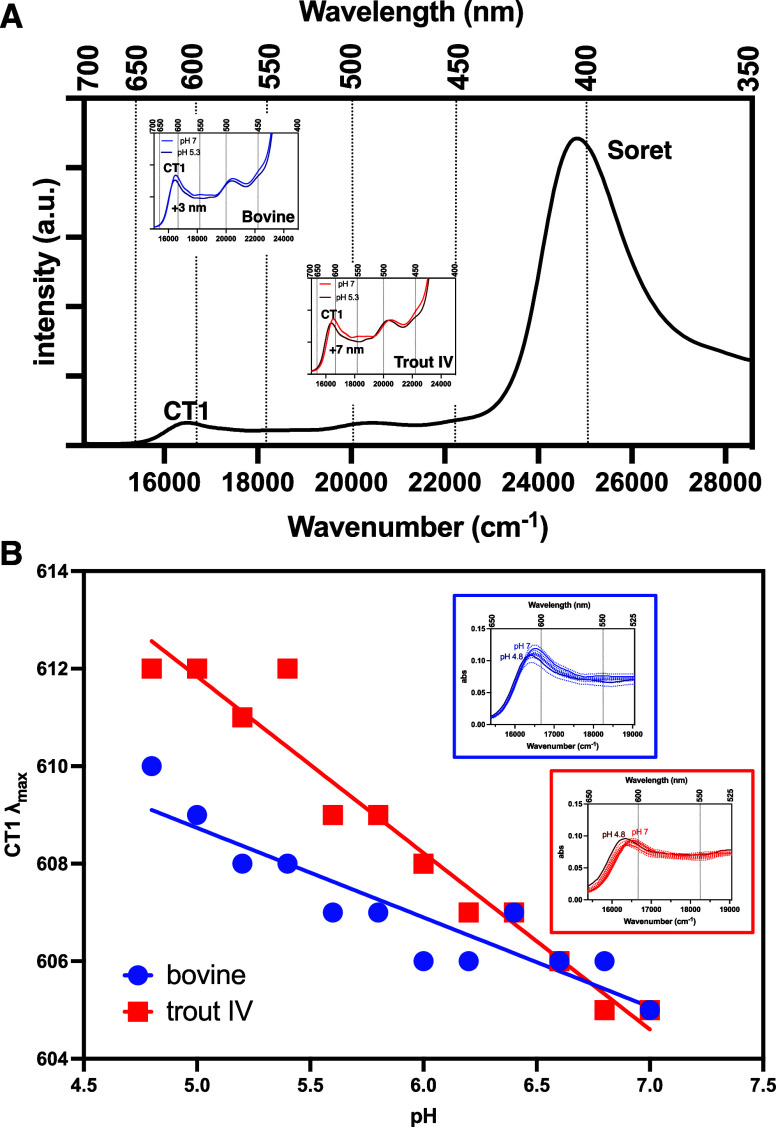
Electronic absorption spectra of metHb-F of trout IV and bovine at different pH values. (A) Spectra of bovine and trout IV metHb-F at low and high pH values. Samples contained 200 μM Hb (heme basis). pH 7 buffer: 45 mM HEPES, 30 mM sodium phosphate, 0.25 mM Tris, and 360 mM NaF. pH 5.3 buffer: 80 mM MES, 50 mM sodium phosphate, 0.25 mM Tris, 360 mM NaF. (B) CT1 red shift as a function of pH. The pH values 7.0–6.2 were assessed using buffer composed of 45 mM HEPES, 30 mM sodium phosphate, 0.25 mM Tris, and 360 mM NaF. The pH values 6.0–4.8 were assessed using buffer composed of 80 mM MES, 50 mM sodium phosphate, 0.25 mM Tris, and 360 mM NaF. Bovine and trout IV metHb were added at 10 μM (heme basis). Electronic absorption spectra were collected at room temperature from 650 to 500 nm. The inset displays the spectral shift with decreasing pH. Data presented are from electronic absorption spectra collected from a single analysis (*n* = 1).

To further confirm a higher p*K*_a_ of the trout IV Hb distal His, we analyzed the metHb-F species by rR spectroscopy at 77 K and pH 5.3 and 7.0 (Figure S1). At pH 7.0 and using λ_ex_ at 457.9 nm, we observed νFe–F values at 471 and 470 cm^–1^ for trout IV and bovine metHb-F, respectively (Figure 3A top). This agrees well with our pH titration (Figure 2B), which indicated that at pH 7, the CT1 transition is at the same wavelength; therefore, it is likely that the strength of the H-bond donation from the distal His is the same for bovine and trout IV Hb at pH 7.0. Further, at pH 7.0, we resolved 7 heme-related vibrational modes (Figure 3A bottom, E), which showed minor differences between trout IV and bovine metHb-F. When using λ_ex_ at 457.9 nm to analyze the pH 5.3 samples, we observed a broad peak at 433 cm^–1^ only for the trout IV metHb-F, which we tentatively assigned as the νFe–F at low pH (Figure 3B top). We also observed the same 7 well-resolved heme peaks (Figure 3B bottom, E) as seen in the high pH sample. To further enhance the weak νFe–F signal, we changed the λ_ex_ to 514.5 nm, which produced a much more intense, broad peak at 428 cm^–1^ that was only observed in trout IV metHb-F (Figure 3C top). Further, at low pH with λ_ex_ = 514.5 nm, we resolved 2 heme pyrrole stretching modes (ν_4_, ν_41_)^56^ and an in-plane C–H bending mode of the vinyl moiety (δC_a_Η=).^56^ These heme-related vibrational modes (δC_a_H=, ν_4_, ν_41_) were observed in both the bovine and trout IV metHb-F spectra (Figure 3C bottom, E). Figure 3D presents heme atom annotations for the interpretation of the vibrational modes presented in Figure 3E.

**Figure 3:**
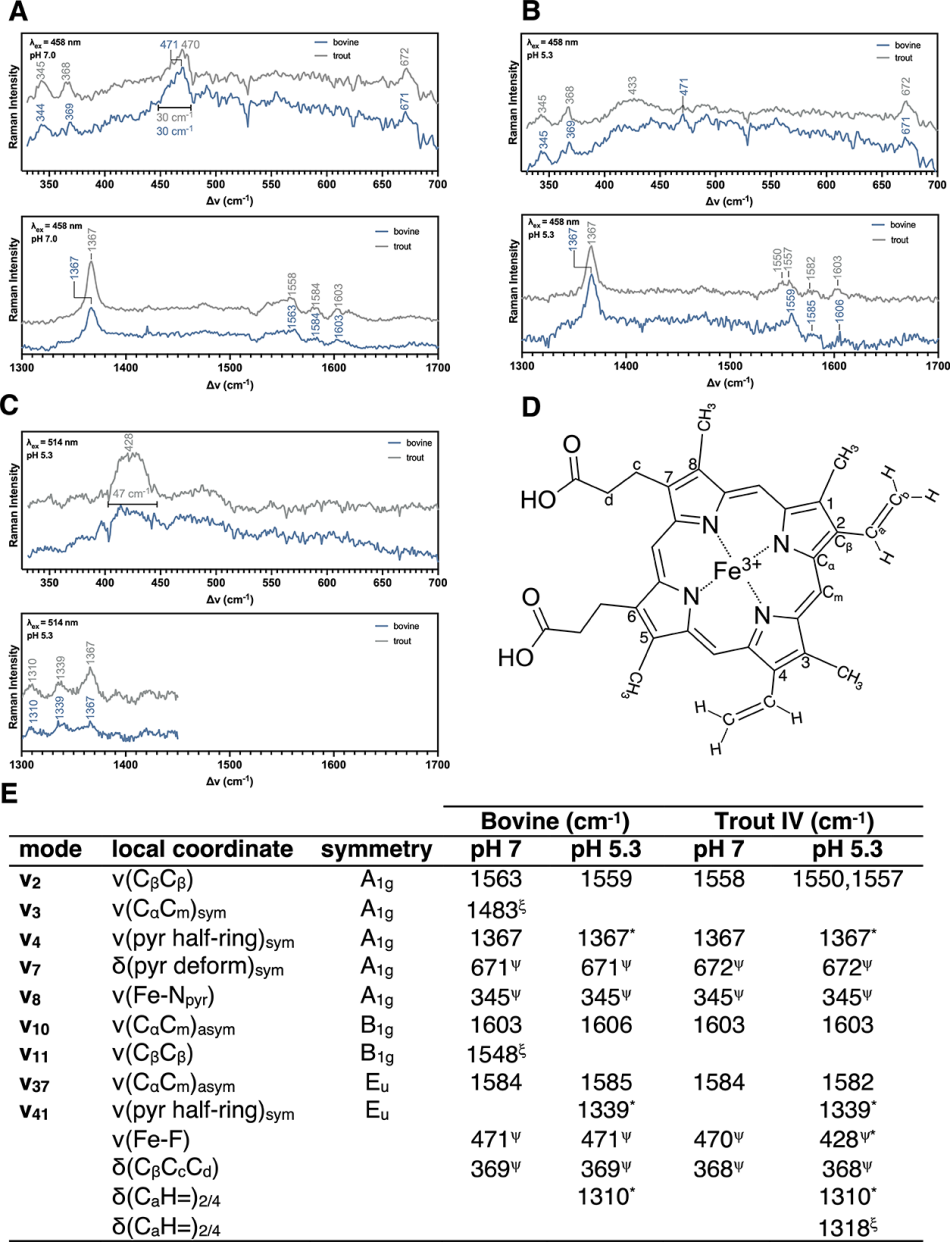
Resonance Raman spectra of bovine and trout IV metHb-F at near physiological pH and post-mortem pH. (A) rR spectra at pH 7.0 (λ_ex_ = 458 nm). Buffer was composed of 45 mM HEPES, 50 mM sodium phosphate, 0.25 mM Tris, and 360 mM NaF. Trout IV data were collected with 19 mW power (at sample); collection times were 820 and 420 s to resolve the low- and high-energy regions, respectively. Bovine data were collected with 22 mW power, and collection times were 1080 and 720 s for low- and high-energy regions, respectively. (B) rR spectra at pH 5.3 (λ_ex_ = 458 nm). Buffer was composed of 80 mM MES, 50 mM sodium phosphate, 0.25 mM Tris, and 360 mM NaF. Trout IV data were collected with 21 mW power, and the collection time was 900 s for both high- and low-energy regions. Bovine data were collected with 22 mW power, 1050 cm^–1^ center, 600 s collection time for both high- and low-energy regions. (C) rR spectra at pH 5.3 (λ_ex_ = 514.5 nm). Buffer composition was the same as that in panel (A). Trout IV data were collected with 21 mW power, 850 cm^–1^ center, 900 s collection time. Bovine data were collected with 22 mW power, 850 cm^–1^ center, 600 s collection time. (A–C) All spectra are the average of 3 readings, with a linear baseline correction applied for the viewing window (e.g., 325–700 cm^–1^). All data were collected at 77 K. All rR data were collected by using a 200 μM Hb concentration (heme basis). (D) Atom labeling of iron protoporphyrin IX. (E) Stretching frequency assignments of experimental rR data. (^ξ^)­Stretching modes only observed in metHb (6th ligand = H_2_O) under our experimental conditions. (^ψ^) Stretching modes only observed in metHb-F under our experimental conditions. (*) Indicates stretching frequencies observed using λ_ex_ = 514.5 nm. All other frequencies were observed using λ_ex_ = 457.9 nm. Stretching frequencies were assigned based on previously published data.^56,72^ Raman data below 300 cm^–1^ were not reported due to the presence of an intense, low-energy feature associated with the ice crystal lattice.

To confirm that the peak observed ca. 430 cm^–1^ was a result of fluoride binding, we also collected rR spectra for nonfluorinated aquometHb samples, with identical buffer systems but without NaF. For pH 5.3, we collected data for trout IV aquometHb at λ_ex_ = 514.5 nm and saw no peak at ∼430 cm^–1^, as expected, and observed 3 high-energy, heme-related stretching modes (Figure S2). For pH 7.0, we collected data for bovine aquometHb at λ_ex_ = 496 nm and again did not see a peak at ∼470 cm^–1^ but were able to identify 4 high-energy, heme-related stretching modes (Figure S3). We were unable to resolve heme stretches for the bovine aquometHb sample at all other excitation wavelengths used (457.9, 514.5, and 496 nm), even when it was very apparent, based on color, that there was heme in the corresponding samples (Figure S1, middle).

To complement our experimental rR findings, we performed DFT calculations to confirm that increasing the H-bond donation (distal His to F^–^) leads to a decrease in νFe–F. We computationally modeled three high-spin, metHb-F models with the distal His in its HSD, HSE, and HSP forms (Figure 4A,B). Notably, as the H-bond donation increases (HSD < HSE < HSP, as measured by the decrease in F–H_ε1_ distance), the Fe–F bond length increases, the proximal N_ε_–Fe bond length decreases, and the core size (R_Ct‑N_) decreases (Figure 4C). It should also be noted that when the distal imidazole is in the HSD state, N_ε1_ rotates away from the fluoride ligand, making H_δ2_ the nearest hydrogen atom to the fluoride ligand. On the basis of these geometric differences, we would expect the computed νFe–F to decrease in the order of HSD > HSE > HSP. The frequency calculations support this hypothesis, with a computed vFe–F of 504.3 cm^–1^ for the HSD model, 445.8 cm^–1^ for HSE, and 388.2 cm^–1^ for HSP (Figure 5). In general, as the H-bond donation increases, mixing of the Fe–F stretching motion with other modes becomes more pronounced. Specifically, for the HSD and HSE models, the vFe–F is relatively pure and nearly perpendicular to the heme plane, while for the HSP model, the assigned vFe–F is a mixture of stretching/bending motions involving the Fe, F, and H_ε1_ atoms (Figure 5, inset).

**Figure 4:**
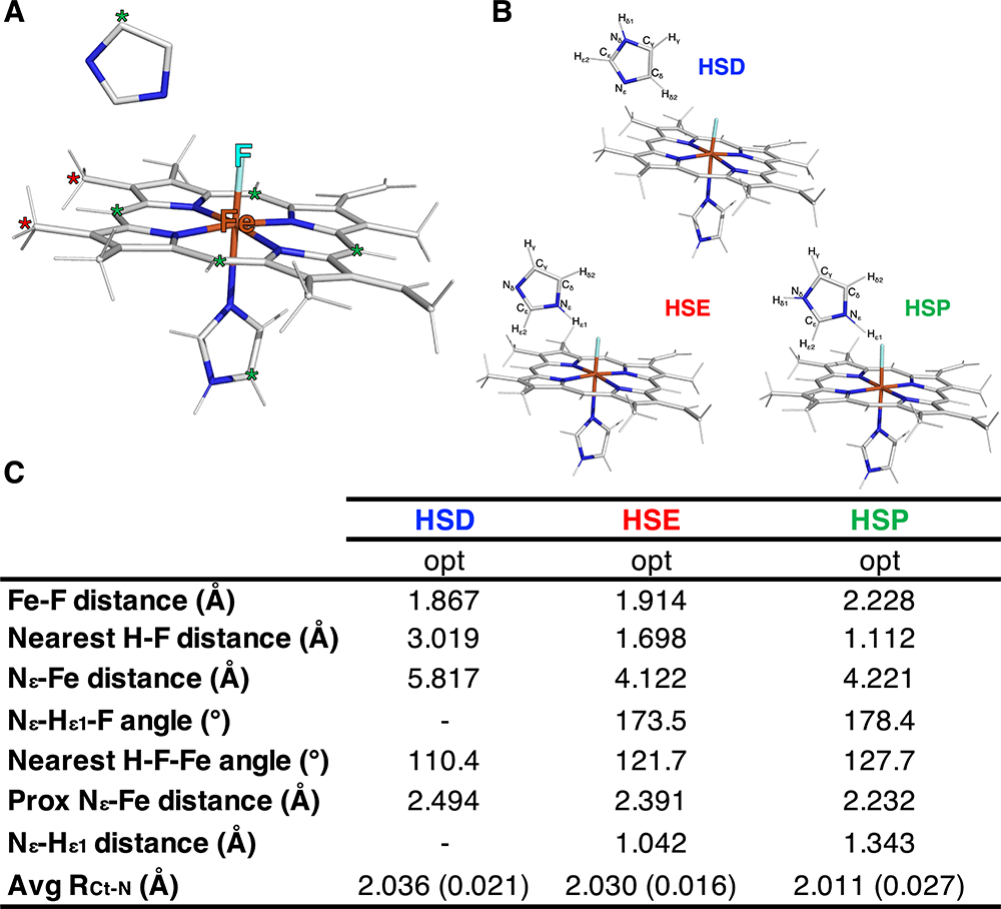
DFT geometry optimized heme models. (A) Annotated heme model. Red asterisks indicate the location of the propionate truncation. Green asterisks indicate the 6 carbons that were fixed during optimization to prevent spurious imidazole-heme plane angles. (B) Optimized structures of heme models. Distal imidazole residues have been assigned atom labels. (C) Bond lengths and angles of optimized heme models with the distal imidazole in different protonation states. R_Ct‑N_ is the average bond distance between the heme iron center and the 4 pyrrole nitrogens (standard deviation in parentheses).

**Figure 5:**
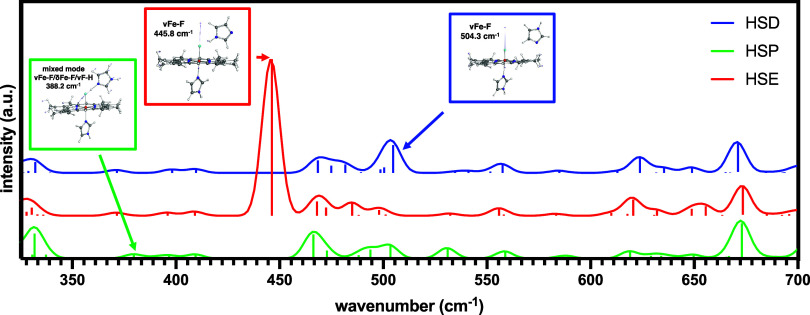
Low-energy-computed Raman spectra for heme models with distal imidazoles of various protonation states (HSD = H atom on N_δ_; HSE = H atom on N_ε_; HSP = doubly protonated). The inset displays the displacement vectors for νFe–F and corresponding vibrational frequencies (cm^–1^) for each of the heme models. Vertical lines display the intensity of vibrational modes contributing to a given spectral peak.

While our experiments utilizing metHb-F proved valuable for probing the distal His H-bond donation at pH 7 and 5.3, in muscle foods, we would not expect substantial fluoride ligation. Therefore, we also sought to characterize ferric Hb in its most biologically relevant and pro-oxidative aquomet (metHb) form. Trout IV metHb displays a unique EA spectrum compared to bovine metHb at pH 5, 6, and 7, most notably for the CT2, low-spin (LS), and CT3 transitions (Figure 6A). These spectral differences are likely caused by an increased LS ferric heme population in trout IV metHb (Figure 6B). Based on the work of Yonetani et al.,^57^ this LS species presumably stems from bis-His axial ligation rather than the presence of an HO^–^ ligand,[Fn fn3] (Figure 6C) due to its presence at low pH.^58^ This notion was further strengthened when overlaying the TDDFT-computed EA spectra of an LS and high-spin (HS) heme model (Figure 6D). An intense feature in the LS heme model spectrum lies directly between the HS CT2 and CT3 absorption bands, which, in a mixed population, would lead to CT2 and CT3 peak broadening, as seen in the experimental spectra (Figure 6A). Furthermore, the computed spectra indicate a red shift of the Soret band from the HS to the LS model (Figure 6D). In agreement with our computational data, the Soret peak of trout IV metHb is red-shifted by 60–120 cm^–1^ at all pH values evaluated in experimental spectra (Figure 6A, Soret region not shown). This Soret red shift further suggests the presence of an LS, bis-His population.^59^

**Figure 6:**
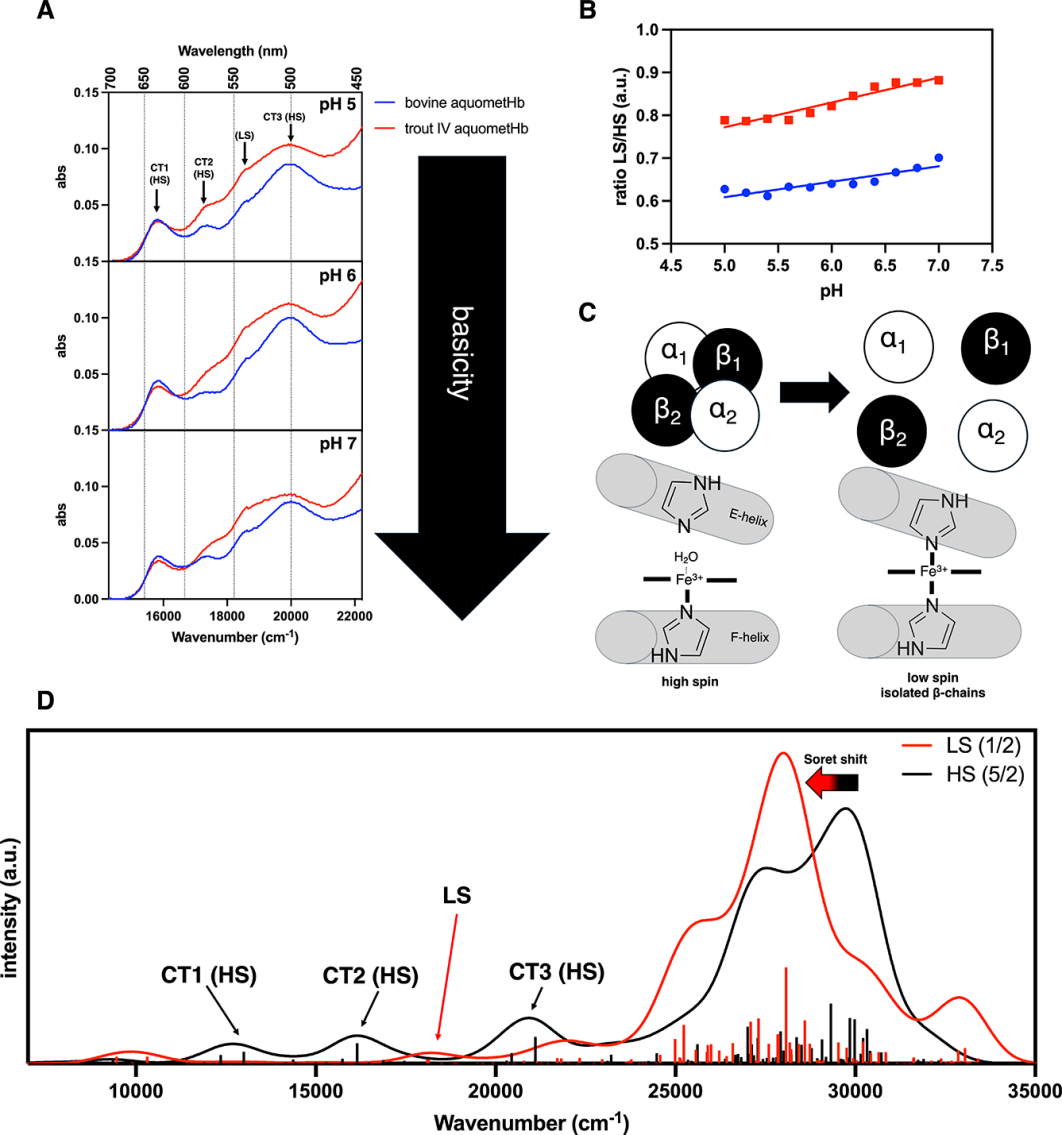
Experimental and computed electronic absorption spectra of bovine and trout IV aquometHb. (A) Annotated experimental electronic absorption spectra of bovine and trout IV aquometHb. Annotations were assigned based on the work of Smith and Williams.^73^ Spectra were collected at room temperature using 10 mM MES as the buffer and 10 μM Ηb (heme basis). (B) Ratio of low-spin (LS) to high-spin (HS) species in bovine and trout IV metHb as a function of pH. Ratio was calculated as follows: abs 18,621 cm^–1^ (537 nm, LS component)/abs 20,000 cm^–1^ (500 nm, HS component).^44^ (C) Schematic of the formation of low-spin ferric Hb observed in trout IV metHb. (D) TDDFT PBE-computed spectra of a ferric, high-spin (5/2), heme model with a 6th water ligand and low-spin (1/2) heme model with proximal and distal imidazole (HSD) residues.

In addition to the His residue in the distal pocket, coordination of the heme iron by the proximal His (Fe–N_prox_ bonding interaction) also plays an important role in hemin release. In the crystal structure of trout IV metHb (PDB ID: 3BOM) at pH 5.7, the average Fe–N_prox_ distance is 2.11 Å compared to 2.08 Å in bovine metHb (PDB ID: 2QSP). Given this difference, we would expect a shift to lower energy for the vFe−Ν_prox_ in trout IV metHb due to the longer bond. To examine the Fe–N_prox_ bond strength by rR spectroscopy, the heme concentration must be high (1–2 mM) and ice crystallization must be prevented to resolve the low-energy (∼230 cm^–1^) Fe–N_prox_ stretch.^38^ To meet these requirements, we prepared metHb rR sample beads in 50:50 (v/v) glycerol:aqueous buffer, with a heme concentration of ∼1 mM (Figure S1, right). Using a λ_ex_ of 496 nm, we were unable to detect the low-energy νFe–N_prox_ in either the trout IV or bovine metHb rR spectra, with only one poorly resolved heme stretching mode (ν_4_ at 1367 cm^–1^) (Figure S4). Similar results were obtained for λ_ex_ values of 514.5 and 457.9 nm (data not shown). Based on DFT calculations, the νFe–N_prox_ stretching mode should be observed at 210–220 cm^–1^ depending on the bond length (Figure S5).

## Discussion

Extensive evidence confirms the increased auto-oxidation,^29^ hemin release,^29,60^ and lipid peroxidation^19,61^ capacity of trout IV Hb compared to mammalian Hb. The data presented in this work provide a framework for interpreting these differences.

Our first finding was that trout IV metHb distal His has a higher p*K*_a_ than bovine metHb. One driving force of this higher p*K*_a_ is the neighboring αTrpCE4, which would form a more stable π–cation interaction than that provided by αPheCE4 of bovine, following HisE7 protonation.^62^ Another driving force would be the substitution of positively charged LysE10 in bovine Hb for ThrE10 in trout IV Hb. LysE10 is within 5 Å of HisE7 and would favor the HSE/HSD form of HisE7 to minimize electrostatic repulsion, thus decreasing the p*K*_a_ of bovine Hb. Based on our rR and computational data (Figures 3–5) for metHb-F, it is likely that at high pH, the distal His residues of both bovine and trout IV Hb are in the HSE form. The HSD form is ruled out upon analysis of our computational results, which show that the negatively charged N_ε_ (−0.37 e) rotates away from the fluoride (−0.44 e), so as to position the H_δ2_ (0.05 e) closest to the F atom in the optimized structure (Figure 4). Further, there is no probable H-bonding partner in the vicinity of the distal His to favor the HSD protonation state over the HSE state at high pH. These results are in good agreement with a previously published report,^63^ which provided evidence that the most likely His protonation state allows for the formation of a H-bond with the fluorine ligand. At low pH (5.3), particularly in trout IV Hb, it is proposed that the distal His is in its HSP protonation state. Interestingly, it seems that the νFe–F for the low pH sample (distal His = HSP) is due to a heterogeneous population of metHb-F species, as shown by the increased peak width (47 cm^–1^), compared to the peak width of νFe–F at high pH (30 cm^–1^). Inspection of the trout IV Hb crystal structure (3BOM, crystallized at pH 5.7) reveals differences in the N_ε_–Fe bond lengths among the 4 chains; however, this is an unlikely contributor to the wide range of νFe–F at low pH because this effect should also manifest itself at higher pH. Instead, the cause of the variable νFe–F at low pH is attributed to variations in the H-bonding network between the nearest heme propionate and the protonated distal His of the α and β chains of trout IV Hb. Crystallographic data (3BOM) suggest that the α-chain heme propionate forms a H-bond network with HisCE3 and TrpCE4, whereas the β-chain heme propionate does not participate in H-bonding at the CD-turn, increasing the likelihood of the heme propionate to H-bond with H_δ1_ of protonated distal His, and influence the νFe–F. These diverging H-bond networks of the heme propionate may drive the formation of multiple metHb-F populations, as evidenced by the increased νFe–F peak width at low pH. One apparent discrepancy between our computational and experimental data of metHb-F is the disagreement between the computed R_Ct‑N_ and the experimental ν_2_ and ν_3_ values. As R_Ct‑N_ decreases with increasing H-bond donation (Figure 4C), an increase in ν_2_ and ν_3_ values should be observed; however, we saw inconclusive changes, probably due to the low signal intensity for the Raman features in the high-energy region (Figure 3E).

Our second important finding is that a larger LS heme population exists in trout IV metHb. Experimentally, this was shown by a red-shifted Soret band in the EA spectrum, broadening of the CT transitions, and an elevated ratio of LS/HS heme species (Figure 6A,B). Computationally, we observed a red-shifted Soret peak and an LS transition within the CT region of the HS EA spectrum, which may lead to experimental peak broadening (Figure 6D). The increased fraction of LS species in the more pro-oxidated trout IV metHb is unexpected. LS species tend to display a decreased ability to activate oxidants (i.e., H_2_O_2_)^64^ and suppress the formation of hypervalent heme. Given this, we would anticipate trout IV metHb to display less LS character than bovine metHb; however, the opposite is true.

Previous studies revealed that isolated β-chains of Hb contain a significant fraction of an LS heme species between pH 5–7.^57^ This LS species was identified as a bis-His-ligated ferric heme (Figure 6C), which formed following a disruption of the Hb α-β interfacial contacts. Based on distances between the H-bond donor and acceptor, trout IV Hb contains residues that form a weaker H-bond network at the α-β tetramer interface, compared to bovine Hb [e.g., αSerC1 (Tyr in bovine), AlaC6 (Thr in bovine)].^65,66^ Therefore, the trout IV Hb tetramer may be more prone to oxidation-induced bis-His formation along the Hb disassembly pathway.^67,68^ Disruption of the tetramer architecture can have major pro-oxidative and functional consequences: (i) Isolated monomers display a significant increase in hemin dissociation;^69^ (ii) individual Hb subunits display increased affinity for lipid membranes compared to a tetramer;^70,71^ and (iii) in some cases, bis-His formation is a prerequisite for protein aggregation.^67^ Hence, despite an unexpected LS population in trout IV metHb, this LS heme population may reflect oxidation-induced tetrameric lability and early-stage disassembly.

The data presented herein lead us to a working model of the pro-oxidative capacity of trout IV metHb, which is shown in Figure 7. Our data suggest that the trout IV Hb distal His presents a higher p*K*_a_, which, upon protonation, particularly in the α-chains, can swing out toward the CE-turn forming a strong cation−π interaction with TrpCE4 (PheCE4 in bovine),^62^ essentially “opening the door” to the heme crevice. It has been well established that when the H-bond between the distal E7 residue and the coordinating ligand is removed, water occupancy decreases,^22,23^ and hemin loss may increase >10-fold.^25^ All of the while, the labile nature of the trout IV Hb global architecture promotes bis-His formation and disassembly.

**Figure 7:**
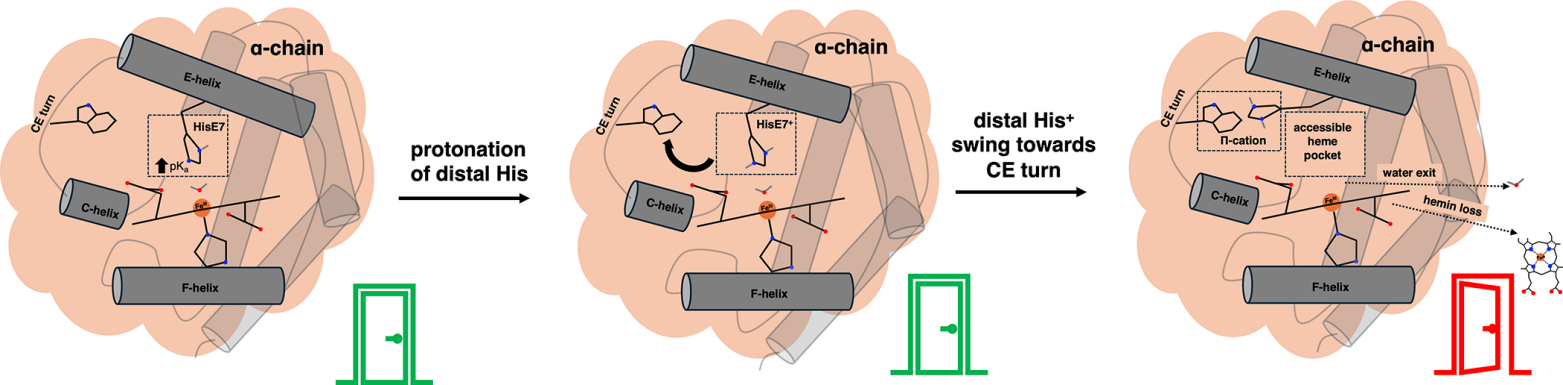
Visual representation of hemin dissociation in trout IV aquometHb. Helices and turns are represented for visualization only and are not drawn to scale.

## Supplementary Material





## Footnotes

1

$r=\frac{\Delta A}{\Delta {Α}_{\infty }}$ . Δ*A* is the abs_607nm_ at 0 mM NaF – abs_607nm_ at X mM NaF. Δ*A*_∞_ is the abs_607nm_ at 0 mM NaF - abs_607nm_ at saturating NaF.

2

$r=\frac{[\mathrm{NaF}{]}^{h}}{K_{D}+[\mathrm{NaF}{]}^{h}}$ . *h* is the hill slope, *K*_D_ is the dissociation constant, and *r* is the fraction metHb-F based on the fitted model.

3 It has been shown that LS ferric bis-his heme species will readily bind fluoride;^58,59^ therefore, the presence of LS species should not impact the results (rR and DFT) obtained from fluorinated heme.

## Notes

Full frequency and TDDFT inputs (XLSX)
Orca calculations inputs; rR sample images (Figure S1); trout IV aquometHb rR control sample (Figure S2); bovine aquometHb rR control sample (Figure S3); rR spectra of trout IV and bovine aquometHb in vitrified media (Figure S4); and DFT-computed proximal His–iron stretching frequency (Figure S5) (PDF)

## Abbreviations

rR: resonance Raman
DFT: density functional theory
TDDFT: time-dependent density functional theory
ν: stretching mode
δ: bending mode
Hb: hemoglobin
Mb: myoglobin
metHb-F: ferric Hb with sixth fluoride ligand
aquometHb/metHb: ferric Hb with sixth water ligand
His: histidine
HSE: histidine with proton on N_ε_
HSD: histidine with proton on Nδ
HSP: positively charged histidine residue with protons on N_ε_ and N_δ_
