# Supplementary material for: Resonance Raman Spectroscopy and Density Functional Theory Reveal the Hemin Release Mechanism of Fish and Mammalian Hemoglobin
Source: J Agric Food Chem. 2026 Feb 20;74(8):7024–33. doi: 10.1021/acs.jafc.5c12506 (PMC12964545; doi:10.1021/acs.jafc.5c12506)
Supplement: Supplementary file 2 [file jf5c12506_si_002.pdf]

Supporting Information

Resonance Raman Spectroscopy and Density Functional Theory Reveal Hemin Release Mechanism of Fish and Mammalian Hemoglobin.

Sean M. Baker[1*], Ryan L. Hall[2], Thomas C. Brunold[2], and Mark P. Richards[1,3]
[1]Department of Food Science, University of Wisconsin–Madison, Madison, WI 53706, USA
[2]Department of Chemistry, University of Wisconsin–Madison, Madison, WI, 53706, USA
[3]Department of Animal and Dairy Sciences, University of Wisconsin–Madison, WI, 53706, USA

[*]Corresponding author. Tel.: +1 507-481-7384

[*]*Email Address: sbaker8@wisc.edu*

Methods Orca Calculations Inputs
Figure S1. rR sample images.
Figure S2. Trout IV aquometHb rR control sample.
Figure S3. Bovine aquometHb rR control sample.
Figure S4. rR of trout IV and bovine aquometHb in vitrified media.
Figure S5. DFT computed proximal his-iron stretching frequency.

Orca Calculation Inputs

<u>Fluoride HSD/HSE/HSP optimization inputs</u>

```
! Opt UKS PBE def2-SVP def2/J  VeryTightSCF NormalPrint D3

%pal nprocs 8 end

%scf  maxiter 300 damp fac 0.85 erroff 0.005 end
end

%geom Constraints
        { C 0 C }
        { C 9 C }
        { C 27 C }
        { C 28 C }
        { C 29 C }
        { C 30 C }
        end
end

*xyz 0 6 #NOTE: charge for HSE and HSD model is 0, charge for HSP model is 1
```

Coordinates can be found on the attached excel file (Sheet *HSD opt, HSE opt, HSP opt*)

```
*
```

<u>Fluoride HSD/HSE/HSP frequency inputs</u>

```
! NumFreq UKS PBE def2-SVP def2/J  VeryTightSCF NormalPrint D3

%pal nprocs 32 end
%elprop Polar 1 end

%scf  maxiter 300 damp fac 0.85 erroff 0.005
end
end

* xyz 0 6 #NOTE: charge for HSE and HSD model is 0, charge for HSP model is 1
```

Coordinates can be found on the attached excel file (Sheet *HSD freq, HSE freq, HSP freq*)

```
*
```

<u>HS Water HSD optimization input</u>

```
! Opt UKS PBE def2-SVP def2/J  VeryTightSCF NormalPrint D3
```

```
%pal nprocs 6 end
%scf  maxiter 300 damp fac 0.85 erroff 0.005 end
end
%geom Constraints
	{ C 0 C }
	{ C 9 C }
	{ C 27 C }
	{ C 28 C }
	{ C 29 C }
	{ C 30 C }
	end
end

*xyz 1 6
```

Coordinates can be found on the attached excel file (Sheet *HS Water opt*)

```
*
```

HS Water HSD optimization scan input

```
! Opt UKS PBE def2-SVP def2/J  VeryTightSCF NormalPrint D3

%pal nprocs 8 end
%scf  maxiter 500 damp fac 0.85 erroff 0.005
end
end
%geom Scan
B 13 52 = 2.0289, 2.3000, 12
end
end
%geom Constraints
	{ C 0 C }
	{ C 9 C }
	{ C 27 C }
	{ C 28 C }
	{ C 29 C }
	{ C 30 C }
	end
end

*xyz 1 6
```

Coordinates can be found on the attached excel file (Sheet *HS Water scan*)

```
*
```

HS Water HSD frequency input

```
! NumFreq UKS PBE def2-SVP def2/J  VeryTightSCF NormalPrint D3

%pal nprocs 32 end
%elprop Polar 1 end

%scf  maxiter 500 damp fac 0.85 erroff 0.005
end
end

*xyz 1 6
```

Coordinates can be found on the attached excel file – inputs slightly changed with increasing Fe-N_{prox} distance (Sheet *HS Water freq 2.1275, HS Water freq 2.1521, HS Water freq 2.1768*)

```
*
```

HS Water HSD TDDFT input

```
! UKS PBE def2-SVP def2/J VeryTightSCF NormalPrint D3 SlowConv RIJCOSX def2-SVP/C

%pal nprocs 8
end

%tddft
        NRoots 160
    MaxDim 5
    EWin   -3,3
    TDA    true
    triplets false
    ETol   -1
    RTol   1e-4
    end

%scf  maxiter 500 damp fac 0.85 erroff 0.005 end
   end

*xyz 1 6
```

Coordinates can be found on the attached excel file – coordinates corresponded to an Fe-N_{prox} distance of 2.1275 ang (Sheet *HS Water TDDFT*)

```
*
```

LS bis-HSD Optimization input

```
! Opt UKS PBE def2-SVP def2/J  VeryTightSCF NormalPrint D3

%pal nprocs 6 end
```

```
%scf  maxiter 500 damp fac 0.85 erroff 0.005 end
end

*xyz 1 2
```

Coordinates can be found on the attached excel file (Sheet *LS bis-HSD opt*)

```
*
```

LS bis-HSD TDDFT input

```
! UKS PBE def2-SVP def2/J VeryTightSCF NormalPrint D3 SlowConv RIJCOSX def2-SVP/C

%pal nprocs 8
end

%tddft
         NRoots 160
      MaxDim 5
      EWin   -3,3
      TDA    true
      triplets false
      ETol   -1
      RTol   1e-4
      end

%scf  maxiter 500 damp fac 0.85 erroff 0.005 end
     end

*xyz 1 2
```

Coordinates can be found on the attached excel file (Sheet *LS bis-HSD opt*)

```
*
```

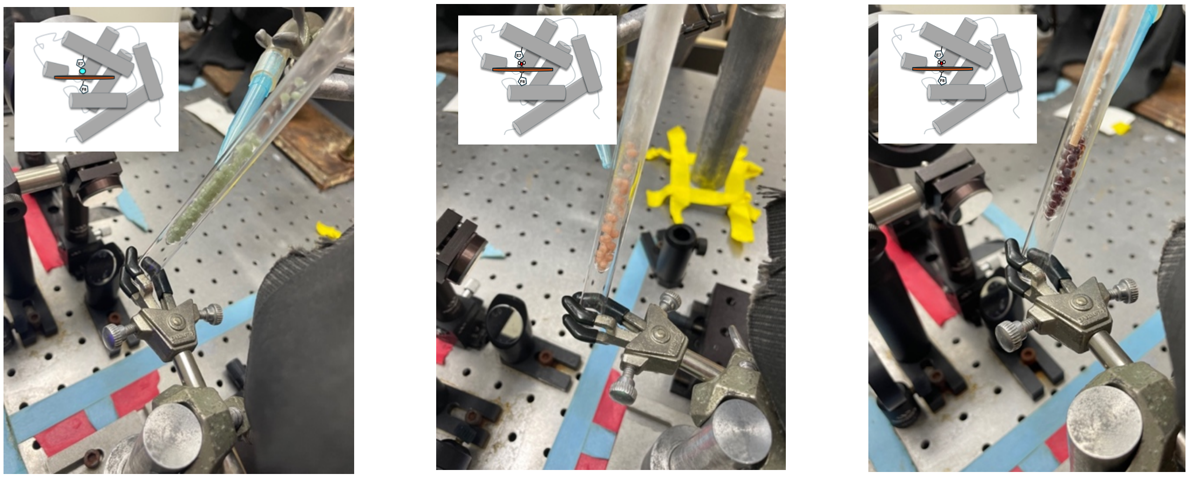

Figure S1. Photo of frozen beads of metHb-F (left, 200 µM heme) in aqueous buffer, aquometHb (middle, 200 µM) in aqueous buffer, aquometHb (right, ~1 mM heme) in 50:50 glycerol: aqueous buffer (vol), loaded into rR sample dewar.

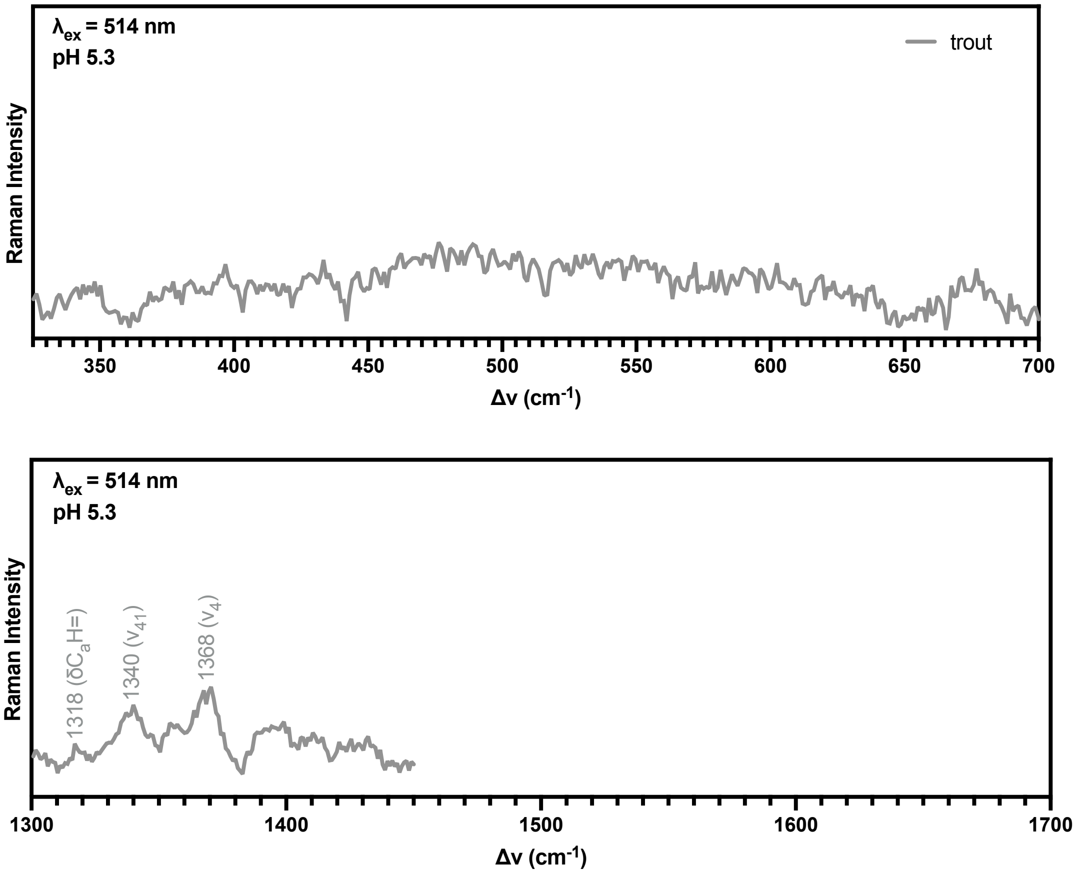

Figure S2. Resonance raman (rR) spectra of trout IV aquometHb (200 µM) pH 5.3 (λ_{ex} = 514 nm). (Top) low energy region, (bottom) high energy region. Buffer was composed of 80 mM MES, 50 mM sodium phosphate, 0.25 mM Tris. Data was collected at 20 mW power (at sample), 850 cm^{-1} center, and 600s collection time, 1read/cycle, 3 cycles. All data was collected at 77K. Raman data below 300 cm^{-1} was not reported due to the presence of an intense, low energy feature associated with the ice crystal lattice.

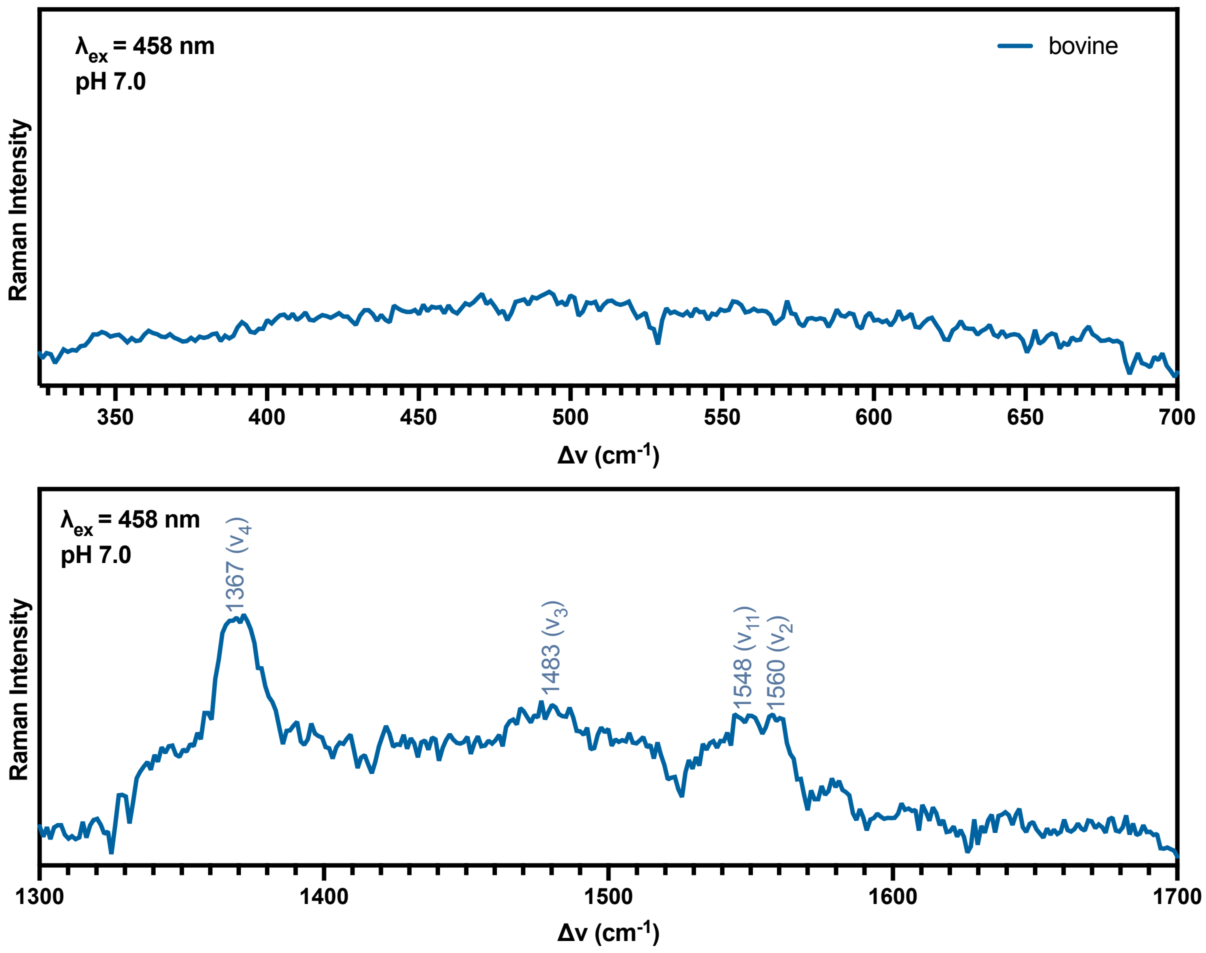

Figure S3. Resonance raman (rR) spectra of bovine aquometHb (400 µM) pH 7.0 (λ_{ex} = 458 nm). (Top) Low energy region, (Bottom) high energy region. Buffer was composed of 45 mM HEPES, 50 mM sodium phosphate, 0.25 mM Tris. Data was collected at 20 mW power (at sample), 1050 cm^{-1} center, and 600s collection time, 1 read/cycle, 3 cycles. All data was collected at 77K. Raman data below 300 cm^{-1} was not reported due to the presence of an intense, low energy feature associated with the ice crystal lattice.

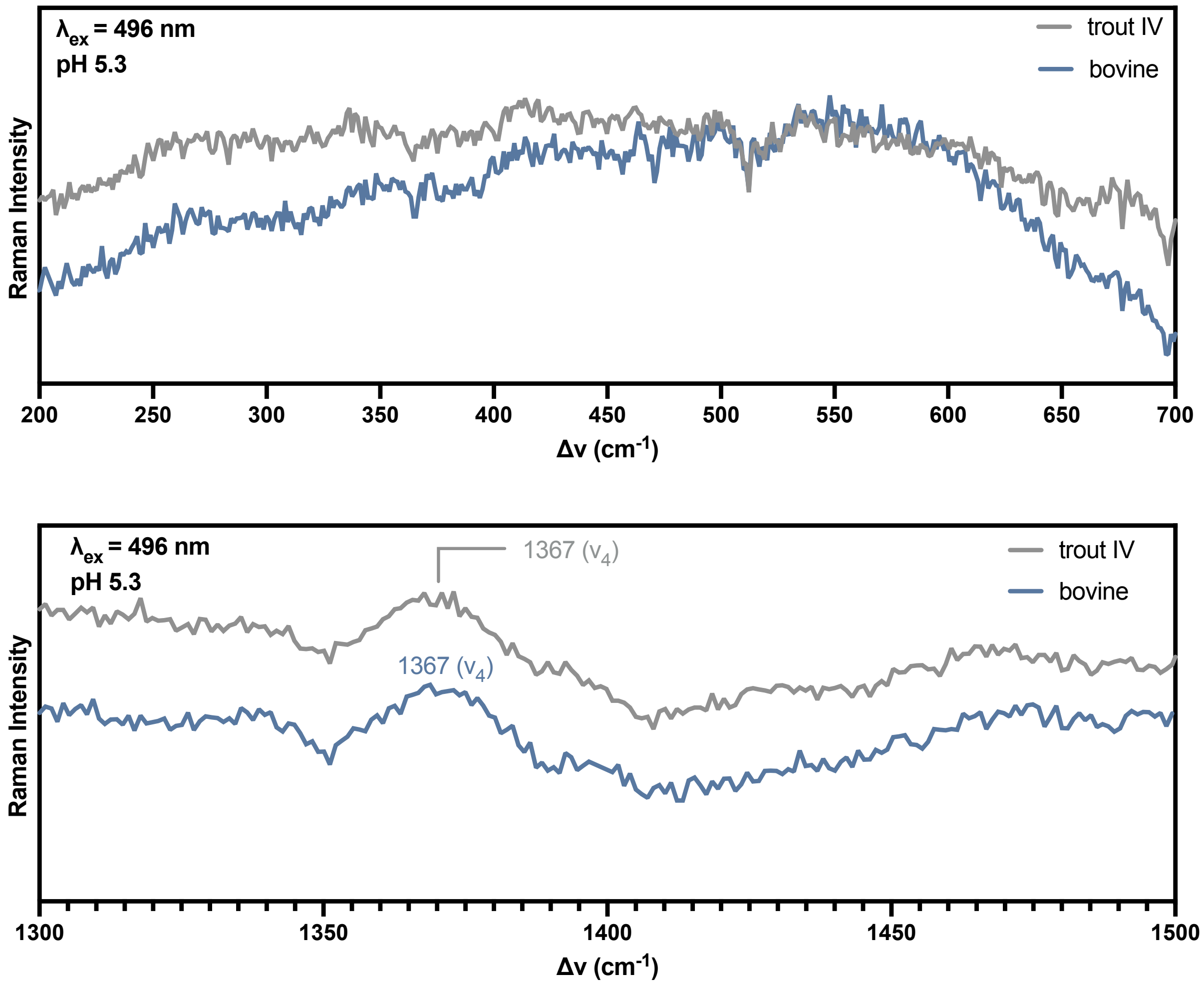

Figure S4. Resonance raman (rR) spectra of bovine and trout IV aquometHb (~1 mM) pH 5.3 (λ_{ex} = 496 nm). (Top) Low energy region, (Bottom) high energy region. Buffer was composed of 80 mM MES, 50 mM sodium phosphate, 0.25 mM Tris. Sample in buffer was combined with glycerol to a ratio of 50:50 (vol). Data was collected at 20 mW power (at sample), 950 cm^{-1} center, and 900s (bovine)/3600s (trout IV) collection time, 1 read/cycle, 1 cycle. All data was collected at 77K. Raman data below 300 cm^{-1} is reported due to sample being prepared in glycerol which eliminates the intense, low-energy ice-lattice associated feature allowing for the viewing of low-energy features.

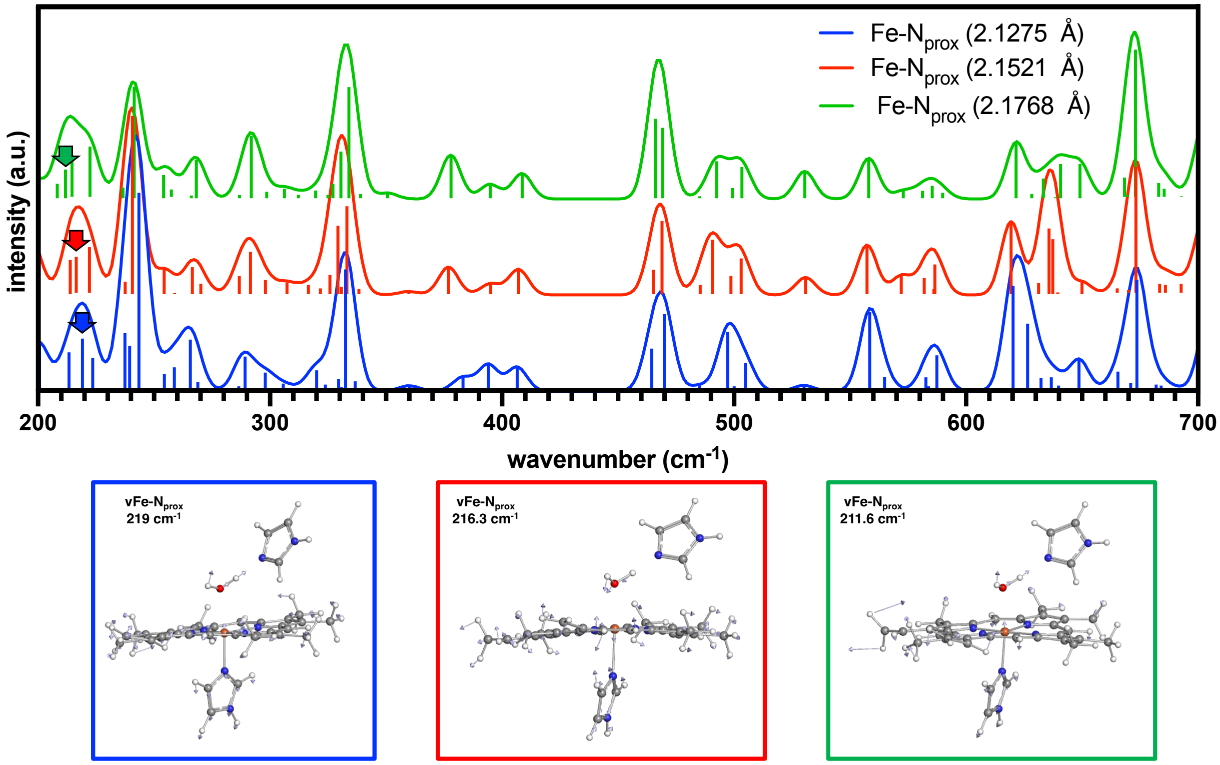

Figure S5. DFT computed low-energy Raman spectra of ferric heme model. The model contains distal and proximal imidazole residues (HSD), a truncated ferric heme, and a 6th water ligand. Spectra were calculated for 3 models with increasing Fe-N_{prox} bond distance (2.1275 Å, 2.1521 Å, 2.1768 Å). The bottom images display the displacement vectors for vFe-N_{prox} and corresponding vibrational frequencies (cm^{-1}) for each of the heme models. Vertical lines display the intensity of vibrational modes contributing to a given spectral peak.
